# FOXO1 promotes the expression of canonical WNT target genes in examined basal‐like breast and glioblastoma multiforme cancer cells

**DOI:** 10.1002/2211-5463.13696

**Published:** 2023-08-28

**Authors:** Shania Pintor, Alma Lopez, David Flores, Brianda Lozoya, Bipul Soti, Rishi Pokhrel, Joaquin Negrete, Michael W. Persans, Robert Gilkerson, Bonnie Gunn, Megan Keniry

**Affiliations:** ^1^ Department of Biology The University of Texas Rio Grande Valley Edinburg TX USA; ^2^ Medical Laboratory Sciences The University of Texas Rio Grande Valley Edinburg TX USA

**Keywords:** basal‐like breast cancer, beta‐catenin, FOXO1, glioblastoma, RNA‐Seq, WNT

## Abstract

Basal‐like breast cancer (BBC) and glioblastoma multiforme (GBM) are aggressive cancers associated with poor prognosis. BBC and GBM have stem cell‐like gene expression signatures, which are in part driven by forkhead box O (FOXO) transcription factors. To gain further insight into the impact of FOXO1 in BBC, we treated BT549 cells with AS1842856 and performed RNA sequencing. AS1842856 binds to unphosphorylated FOXO1 and inhibits its ability to directly bind to DNA. Gene Set Enrichment Analysis indicated that a set of WNT pathway target genes, including lymphoid enhancer‐binding factor 1 (*LEF1*) and transcription factor 7 (*TCF7*), were robustly induced after AS1842856 treatment. These same genes were also induced in GBM cell lines U87MG, LN18, LN229, A172, and DBTRG upon AS1842856 treatment. By contrast, follow‐up RNA interference (RNAi) targeting of *FOXO1* led to reduced *LEF1* and *TCF7* gene expression in BT549 and U87MG cells. In agreement with RNAi experiments, CRISPR Cas9‐mediated *FOXO1* disruption reduced the expression of canonical WNT genes *LEF1* and *TCF7* in U87MG cells. The loss of *TCF7* gene expression in *FOXO1* disruption mutants was restored by exogenous expression of the DNA‐binding‐deficient *FOXO1‐H215R*. Therefore, FOXO1 induces *TCF7* in a DNA‐binding‐independent manner, similar to other published FOXO1‐activated genes such as *TCF4* and hes family bHLH transcription factor 1. Our work demonstrates that *FOXO1* promotes canonical WNT gene expression in examined BBC and GBM cells, similar to results found in *Drosophila melanogaster*, T‐cell development, and murine acute myeloid leukemia models.

## Graphical abstract

FOXO transcription factors are emerging as key regulators of the WNT pathway in settings ranging from osteoblasts to cancer. This work demonstrates for the first time that FOXO1 promotes the expression of WNT pathway target genes *LEF1* and *TCF7* in basal‐like breast and glioblastoma multiforme cells. *FOXO1* RNAi or disruption led to reduced expression of *LEF1* and *TCF7*.

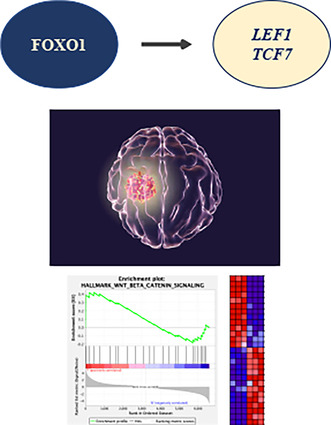

**Abbreviations**
4‐OHT: 4‐hydroxytamoxifen
APC: APC regulator of WNT signaling pathway, adenomatosis polyposis coli
AML: acute myeloid leukemia
Axin2: axis inhibition protein 2
BBC: basal‐like breast cancer
beta 1: (Ctnnb1); catenin cadherin‐associated protein
CCND1: Cyclin D1
CD44: CD44 antigen
CDC42: cell division cycle 42
CDKN1B: cyclin‐dependent kinase inhibitor 1B
CSL: cbf1, suppressor of hairless, lag‐1
DEGs: differentially expressed genes
EVI: ecotropic virus integration site 1 protein homolog
FOXO: Forkhead box O
FPKM: fragments per kilobase of transcript sequence per million base pairs sequenced
GADD45A: growth arrest and DNA damage‐inducible alpha
GBM: glioblastoma multiforme
GSEA: Gene Set Enrichment Analysis
GSK3: glycogen synthase kinase 3
HER2: Human epidermal growth factor receptor 2, Receptor tyrosine‐protein kinase erbB‐2
*HES1*
: hes family bHLH transcription factor 1
HEY1: hes related family bHLH transcription factor with YRPW motif 1
INSR: insulin receptor
*LEF1*
: lymphoid enhancer‐binding factor 1
MYC: master regulator of cell cycle entry and proliferative metabolism
MDS1: myelodysplasia syndrome 1
MMP26: matrix metallopeptidase 26
PROX1: Prospero homeobox protein 1
RNAi: RNA interference
siRNA: small interfering RNA
SOD2: superoxide dismutase 2
TCF4: transcription factor 4
*TCF7*
: transcription factor 7
TRAIL: TNF superfamily member 10
TUBB: beta‐tubulin gene

Basal‐like breast cancer (BBC) and glioblastoma multiforme (GBM) are cancers that are associated with poor prognosis and harbor stem gene expression signatures, which contribute to aggressiveness [1, 2, 3]. GBM is a cancer of the central nervous system and is resistant to conventional chemotherapy [4, 5, 6, 7]. Because of high recurrence and metastatic abilities, GBM is associated with a mean survival time of 8 to 15 months [2]. GBM has intratumor heterogeneity or mutative differences across tumors that make it challenging to target [3, 4, 7]. Basal‐like breast cancer is the most aggressive molecular subtype of breast cancer [8, 9, 10, 11, 12, 13, 14, 15, 16, 17]. BBC rarely responds to hormone‐based therapies due to a lack of estrogen, progesterone, and HER2 receptor expression [9, 17]. BBC is more common in young and African American women [9, 17]. Around 14% of breast cancers are BBC; these cancers are usually advanced at diagnosis [18].

The Forkhead box transcription factor subclass O includes homologs FOXO1, FOXO3, FOXO4, and FOXO6 [19]. FOXO‐1, FOXO‐3, and FOXO‐4 are partially redundant tumor suppressors that induce pro‐apoptotic genes such as *TRAIL* and cell cycle inhibitors such as cyclin‐dependent kinase inhibitor 1B (*CDKN1B*; encoding p27) in some settings [19, 20]. FOXO factors act in a context‐dependent manner and are heavily regulated by post‐translational modifications to direct specific responses [19, 20]. For example, oxidative stress leads FOXO factors to interact with beta‐catenin to induce genes such as *GADD45A* in osteoblasts [21, 22]. FOXO factors also maintain embryonic, hematopoietic, neural, and cancer stem cells [23, 24, 25, 26, 27, 28, 29, 30, 31].

The WNT pathway facilitates stem cell renewal, differentiation, and proliferation during development and adult tissue homeostasis [32]. Canonical WNT pathway targets such as lymphoid enhancer‐binding factor 1 (*LEF1*) and transcription factor 7 (*TCF7*) are activated by beta‐catenin/TCF complexes [33]. In canonical signaling, secreted WNT ligands bind to Frizzled receptors, which ultimately inactivate the APC complex [33]. Loss of APC leads to beta‐catenin stabilization, which triggers transcription of WNT genes [34, 35]. Noncanonical WNT planar cell polarity signaling impacts asymmetric cell patterning to direct orientation of subcellular structures [36, 37, 38, 39]. WNT‐Ca^2+^ signaling promotes actin polymerization via CDC42 activation to increase migration. WNT‐Ca^2+^ also impacts cell fate via activation of nuclear factor of activated T cells and activating transcription factor 2 [40, 41, 42].

In cancer, the WNT pathway promotes proliferation, cell fate, and stem cell function in diverse settings, including colon, breast, and GBM [43]. In BBC, beta‐catenin promotes migration and *MYC* expression [44]. The WNT5A, WNT5B, and WNT7B ligands are highly expressed in BBC, impacting canonical and noncanonical WNT signaling [45, 46, 47, 48, 49, 50]. The WNT pathway affects cell fate and progression in GBM [51, 52, 53, 54, 55, 56, 57, 58]. Activation of the WNT pathway induced autophagy‐mediated temozolomide resistance in GBM [51]. Beta‐catenin was required for cancer stem cell self‐renewal in a subset of GBM patient‐derived models [54].

*Drosophila melanogaster* has a single FOXO protein (dFoxO) that can act with armadillo (beta‐catenin homolog) to pattern wings, eyes, and induce apoptosis. Overexpression of *armadillo* with *sd*‐Gal4 led to an ectopic bristle phenotype near the wing margins in adult flies. Loss‐of‐function *dfoxo* mutants (*dfoxo*‐IR, which had half the normal expression of dFoxO, *dfoxoD94*/+, or *dfoxo21*/+) suppressed the *armadillo*‐driven ectopic bristle phenotype [59]. Furthermore, ectopic gene expression of *wf* (*wingful*, human *NOTUM* homolog) reporter gene *wf‐LacZ* in third instar wing disks and salivary glands was induced by overexpression of *armadillo* (via *ptc*‐Gal4); this phenotype was strongly suppressed by *dfoxo* loss‐of‐function mutants (*dfoxo*‐IR, *dfoxoD94*/+, or *dfoxo21*/+) [59]. In the *Drosophila* eye, overexpression of *armadillo* or *dFoxO* (using GMR‐Gal4) led to a small eye phenotype due to increased apoptosis. Loss‐of‐function mutants in *armadillo* suppressed the small eye phenotype of *dFoxO* overexpression, whereas loss‐of‐function *dfoxo* suppressed the small eye phenotype of *armadillo* overexpression [59]. In addition to numerous genetic interactions observed between *dFoxO* and *armadillo*, these proteins are physically associated. Myc‐tagged armadillo protein associated with FLAG‐tagged dFoxO in co‐immunoprecipitation analyses [59]. FOXO transcription factors can bind to, and act in concert with, beta‐catenin in additional contexts, such as HEK 293T cells, to induce the gene expression of GHOST transcripts (genes hidden outside the standard targets) such as *GADD45A* [60]. In preosteoblasts, FOXO1 and beta‐catenin induce genes such as *GADD45A* in response to oxidative stress [22]. Beta‐catenin‐FOXO1 was also shown to hinder kidney fibrosis [61, 62].

Foxo1 was required for canonical WNT gene expression in osteoblasts and a murine acute myeloid leukemia (AML) model induced by activated beta‐catenin. Forced expression of *Foxo1* led to increased expression of *Axin2* and *Lef1* in murine osteoblasts [63]. Furthermore, constitutively active beta‐catenin (*Ctnnb1* ^*CAosb*^) had increased expression of targets *Axin2* and *Lef1*, which were significantly reduced by osteoblast‐specific deletion of *Foxo1* [63]. In murine osteoblasts, active beta‐catenin (*Ctnnb1* ^*CAosb*^) drives AML development [64]. Osteoblast deletion of *Foxo1* prevented *Ctnnb1* ^*CAosb*^‐driven AML development as evidenced by a lack of blasts and neutrophils with nuclear hypersegmentation in the blood, as well as blast infiltration of liver at periportal sites; this type of liver infiltration is commonly observed in murine AML models driven by active beta‐catenin and fusion proteins AML1/MDS1/EVI [63, 65]. Therefore, evidence points to a role for Foxo1 in driving WNT gene expression in AML development.

There is evidence that FOXO factors impede canonical WNT gene expression in some contexts, including osteosarcoma, pancreatic, and colon cancer cells. FOXO1 tumor suppressor activity inhibited the WNT pathway in osteosarcoma cell lines U2OS and MG63 [66]. *FOXO1* was overexpressed for 48 h using an integrated *FOXO1*‐ER fusion that was induced to go to the nucleus by 4‐OHT treatment; this led to decreased WNT pathway reporter activity using TOPflash (reporter contains TCF/LEF1 sites that regulate luciferase expression) [66]. In addition, FOXO1‐induced cells had less detected beta‐catenin protein based on immunofluorescence. FOXO1 induction using 4‐OHT also led to cell cycle arrest and apoptosis, highlighting canonical FOXO roles in cancer.

FOXO1 hindered the WNT pathway in pancreatic cancer [67]. *FOXO1* was overexpressed using an integrated, selected virally transduced cassette, leading to induction in the gene expression of a long noncoding RNA *LINC01197* in a series of pancreatic cancer cell lines AsPC1, BxPC3, and PANC1 [67]. Reduction in *FOXO1* by siRNA in HPNE (immortalized pancreatic duct cells) led to a loss in *LINC01197* expression. *LINC01197* RNA attached to biotin beads interacted with beta‐catenin protein in RNA pull‐down assays. Overexpression of *LINC01197* for 48 h decreased WNT target gene expression, as measured by quantitative real‐time PCR (qRT‐PCR; *MYC*, *CCND1*, *CD44*, and *MMP26*), and disrupted co‐immunoprecipitation between beta‐catenin and TCF4 (using endogenous proteins) from pancreatic ductal adenocarcinoma cells. Conversely, *LINC01197* siRNA for 48 h increased WNT target gene expression measured by qRT‐PCR (*MYC*, *CCND1*, *CD44*, and *MMP26*, and the interaction between endogenous TCF4 and beta‐catenin in HPNE cells. The authors present a model in which FOXO1 inhibits the TCF4/beta‐catenin interaction in pancreatic cancer via induction of *LINC01197* [67].

FOXO transcription factors have a complex role in the solid tumors BBC and GBM. These factors act as tumor suppressors by activating apoptosis under the right conditions and are pro‐oncogenic by directly inducing stem gene expression [19, 23, 68, 69, 70]. However, the extent to which FOXO transcription factors influence BBC and GBM biology, especially under basal conditions, remains an open area of investigation. In this work, we treated BBC and GBM cells with AS1842856 to discover novel FOXO‐regulated processes. AS1842856 was identified for its ability to specifically bind to the unphosphorylated form of FOXO1, thereby blocking the ability of this factor to directly bind to DNA [71]. A set of WNT pathway target genes were robustly induced by AS1842856 not only in BT549 cells but also broadly in a series of GBM cell lines. In contrast to AS1842856 treatment (which blocks FOXO1 DNA binding), *FOXO1* gene disruption or RNAi targeting led to reduced *LEF1* and *TCF7* gene expression in U87MG cells. Expression of the DNA‐binding‐defective *FOXO1‐H215R* mutant restored *TCF7* gene expression to *FOXO1* disruption mutants. Therefore, the DNA‐binding activity of FOXO1 is not required to induce *TCF7*, similar to other published targets *TCF4* and hes family bHLH transcription factor 1 (*HES1*) [72, 73]. Our work is the first to demonstrate that *FOXO1* promotes canonical WNT gene expression in examined solid tumor‐derived BBC and GBM cells, similar to results found in *Drosophila melanogaster*, T‐cell development, and beta‐catenin‐driven AML models.

## Materials and methods

### Cell lines, cultures, and drug treatments

Cell lines were obtained from ATCC (American Type Culture Collection, Manassas, VA) and grown with 5% CO_2_, 10% fetal bovine serum, and 5% antifungal/antibacterial (Anti/Anti, Thermo Fisher, Waltham, MA, USA). Cell lines were tested for Mycoplasma (Lonza, Basel Switzerland, cat: LT07‐218). U87MG cells were grown in MEM (Minimal Essential Medium). BT549 and DBTRG cells were grown in RPMI (Roswell Park Memorial Institute 1640 Medium). LN18, U118MG, A172, LN229, HCT116, and SW480 cells were grown in DMEM (Dulbecco's Modified Eagle Medium). AS1842856 was purchased from Calbiochem (Danvers, MA) and used at the final concentration of 1 μm for 48 h (except when noted).

### RNAi experiments

BT549 and U87MG cells were transfected with *FOXO1* esiRNA (EHU156591 Sigma, St. Louis, MO), *FOXO3* (EHU113611), *FOXO4* (EHU075731), or EGFP control esiRNA (*EHUEGFP*) using Lipofectamine 3000 (utilized only L3000 reagent, Invitrogen, Carlsbad, CA). We also utilized *FOXO1* RNAi from Cell Signaling Technologies with control (cat: 6242, 6256, and 6568, respectively) and obtained the same results as the Sigma esiRNA.

### FOXO1 disruption

The *FOXO1* gene was disrupted in U87MG cells using the OriGene CRISPR Cas9 knock‐out kit Cat#: KN400477 (Rockville, MD). Mutants were confirmed by western blot and sequencing. The truncated protein is predicted to start in the middle of the DNA‐binding domain and contain the C terminus of the protein.

### Transfection of *FOXO1‐H215R* mutant

One million log‐phase U87MG cells were transfected using a LONZA electroporation kit (VPG1001 for neurons) and program T‐020 with 500 nanograms of indicated DNA (control vector or *FOXO1‐H215R* mutant) [73]. RNA was prepped from cells 24 h post‐transfection.

### *RNA* extraction and sequencing

One μm AS1842856 or (DMSO control) was added to BT549 cells for 48 h. Total RNA was extracted using Qiagen RNAeasy and DNAse reagents (Hilden, Germany). Three biological replicates of control and AS1842856‐treated samples were used for RNA extraction. The quality of the extracted RNA was analyzed using a NanoDrop spectrophotometer (Thermo Fisher, USA) and Agilent Bioanalyzer (Santa Clara, CA).

RNA‐Seq analysis, including mapping to the human genome, sequence assembly, and identification of differentially expressed genes (DEGs), was performed by the Novogene Corporation Inc. (Sacramento, CA). Original image data files from high‐throughput sequencing (Illumina) were transformed into sequenced reads (called Raw Data or Raw Reads) by CASAVA base recognition. star software was used for mapping. FPKM (Fragments Per Kilobase of transcript sequence per Million base pairs sequenced) was utilized to estimate gene expression levels. Differential expression analysis was performed using DESeq2 R package.

RNA‐Seq data were deposited to the GEO database (Accession: GSE179856). RNA‐Seq data were analyzed to identify DEGs associated with AS1842856 treatment. Differentially expressed genes between AS1842856 treated and control cell lines were investigated using Gene Set Enrichment Analysis (GSEA) [74]. GSEA was performed by setting ENSEMBL Gene ID v7.3 Chip platform, 1000 permutations, Signal2Noise, and weighted enrichment statistics. Gene expression box plots from clinical samples were prepared using the gepia 2 online tool [75].

### Quantitative real‐time PCR

Log‐phase cancer cell lines were treated with 1 μm AS1842856 for 48 h (unless otherwise noted). Extracted total RNA was used to prepare cDNA using Superscript Reverse Transcriptase II (Invitrogen, Carlsbad, CA). Primers spanning coding sequences for the genes were designed using Primer3 (v0.4.0) (https://bioinfo.ut.ee/primer3‐0.4.0/) and ordered from Sigma‐Aldrich (Saint Louis, MO). Amplification and expression of genes were performed using the Applied Biosystems StepOne Real‐Time PCR System (Foster City, CA). *TUBB* (*beta‐tubulin* gene) was used as the control for relative normalization and quantification performed by the 2^−ΔΔCT^ method (primer list in Table S1) [76]. The Student's *t*‐test was employed to assess statistical significance.

### Western blot

Cells were rinsed with 1XPBS (phosphate‐buffered saline) followed by lysis in 2X sample buffer (125 mm Tris–HCL at pH 6.8, 2% sodium dodecyl sulfate (SDS), 10% 2‐mercaptoethanol, 20% glycerol, 0.05% bromophenol blue, and 8 m urea); 2X sample buffer was added to each well, samples scraped, and heated for 10 min at 95 °C in a dry‐bath heat block. Protein lysates were resolved by sodium dodecyl sulfate‐polyacrylamide gel electrophoresis (SDS/PAGE and then transferred onto a polyvinylidene fluoride (PVDF) membrane. Blots were blocked in a 5% milk solution [Carnation powdered milk, 1X Tris‐buffered saline with Tween 20 (TBST)] for an hour. Membranes were incubated with indicated primary antibody overnight at 4 °C, washed, and then incubated with a secondary antibody for 1.5 h. Membranes were washed and developed using SuperSignal West Dura Extended Duration Substrate luminol solution (Pierce Biotechnology, Waltham, MA) for 5 min. A Bio‐Rad ChemDoc XRS + Molecular Imager was used for protein detection (Bio‐Rad Hercules, CA). Antibodies were obtained from Cell Signaling Technologies (Danvers, MA) beta‐catenin (cat: 94530), FOXO1 C29H4 (cat: 2880), FOXO3 D19A7 (12829), P‐FOXO1 S256 (cat: 9461), FOXO4 55D4 (cat: 2499), Histone H3 D1H2 (cat: 4499), P‐GSK3alpha/beta (ser 21/9 (cat: 9331), and GSK3 alpha/beta D75D3 (cat: 5676). Alpha tubulin antibody (clone AC‐74, cat: A2228) was obtained from Sigma‐Aldrich (Saint Louis, MO). GAPDH (cat: G‐9) was from Santa Cruz Biotechnology (Dallas, TX).

### Ethics approval

Work was performed with Institutional Biosafety Committee approval from the University of Texas Rio Grande Valley: Registration number: 2016‐003‐IBC.

## Results

### RNA‐Seq revealed AS1842856 treatment increased WNT gene expression in BBC BT549 cells

**Fig. 1:**
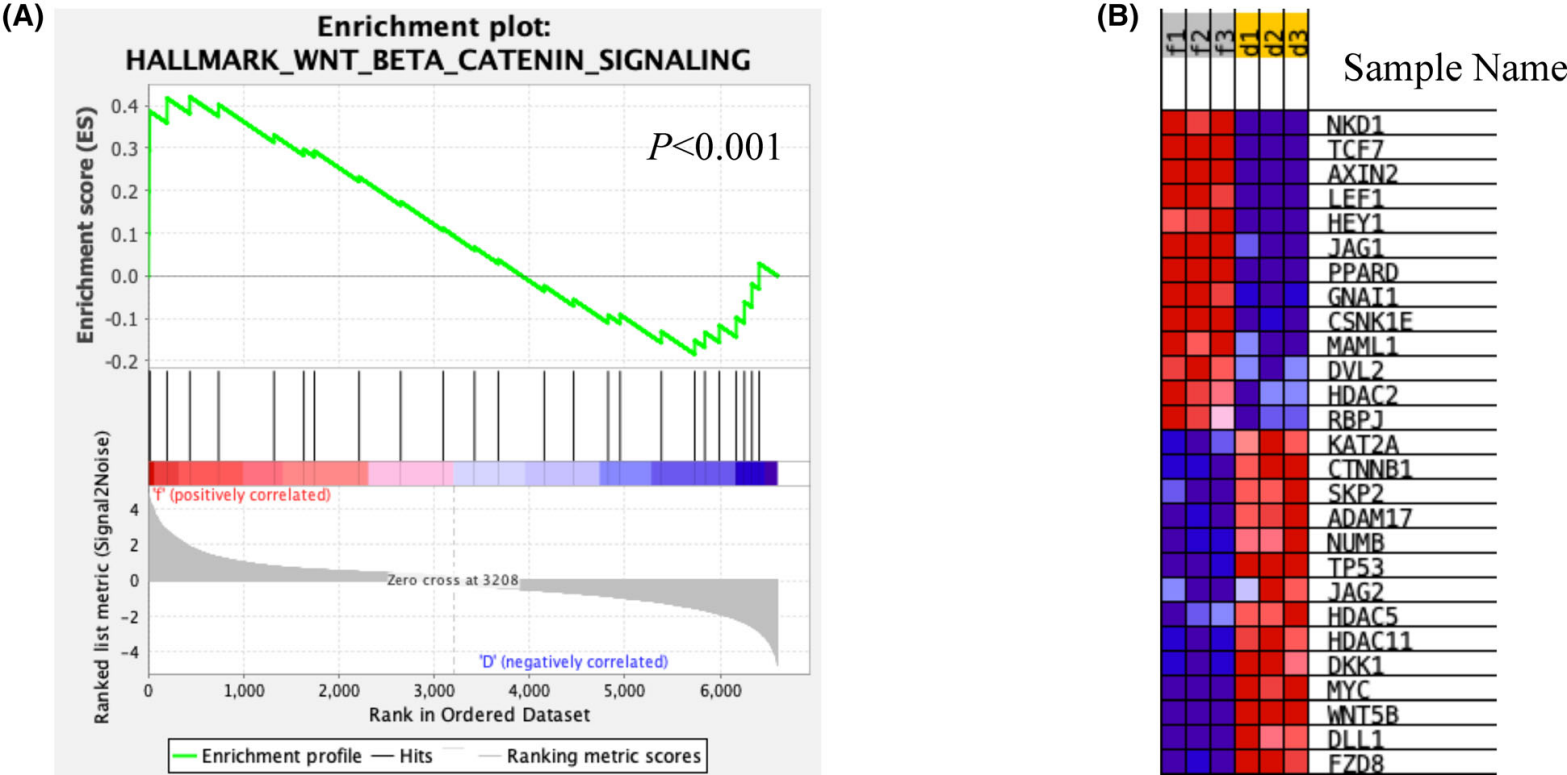
FOXO1 inhibition with AS1842856 induced a set of Hallmark WNT beta‐catenin genes based on GSEA. (A) GSEA was performed with RNA‐Seq data upon 1 μm AS1842856 treatment for 48 h in BT549 cells. Enrichment plot of Hallmark WNT and beta‐catenin. The profile of the running enrichment score (ES) and rank‐ordered list are shown for GSEA enrichment. (B) Heatmap of 27 core‐enriched genes in HALLMARK_WNT_BETA_CATENIN signaling gene set.

FOXO transcription factors reside at least in part in the nucleus in BBC and GBM cells and promote stem gene expression programs [23, 70, 77]. The roles of FOXO factors in BBC and GBM, especially under basal conditions, remained to be fully characterized. To identify FOXO1 target genes (direct and indirect), RNA‐Seq analysis was performed with BT549 cells that were treated with 1 μm AS1842856 (which blocks the ability of FOXO1 to directly bind DNA) for 48 h [71]. Gene expression levels were estimated by the abundance of transcripts (count of sequencing) that mapped to the genome. Differential expression analysis between DMSO control (three biological replicates) and AS1842856 treated samples (three biological replicates) was performed using the DESeq2 R package. 6754 differentially expressed genes (DEGs) were identified. Gene Set Enrichment Analysis was performed to discover processes impacted by AS1842856 treatment. Examining the Molecular Signatures Database Hallmark genesets (h.all.7.4symbols.GMT), we found that two were significantly enriched in AS1842856‐treated samples Table 1, whereas 16 were enriched in the controls (Table 2). Several well‐characterized FOXO1‐induced genes had decreased expression with AS1842856 treatment including *SOD2*, *INSR*, and *GADD45A* (*P*‐values 1.37E‐158, 3.54E‐08, and 1.27E‐21 respectively) [78, 79, 80]. WNT genes (HALLMARK_WNT_BETA_CATENIN) were significantly enriched in the AS1842856‐treated samples compared with controls (*P* < 0.001 with a NES = 1.9) suggesting that AS1842856 treatment induced the expression of a set of WNT genes (Fig. 1A,B).

**Table 1:** Genesets enriched in AS1842856‐treated BT549 samples compared with controls.

Geneset name	ES	NES	NOM *P*‐value	FDR *q*‐value	FWER *P*‐value
HALLMARK_WNT_BETA_CATENIN_SIGNALING	0.42	1.9	*P* < 0.001	0.047	*P* < 0.001
HALLMARK_KRAS_SIGNALING_UP	0.35	1.3	*P* < 0.001	0.348	0.648

**Table 2:** Genesets enriched in BT549 controls compared with AS1842856 treatment.

Geneset name	ES	NES	NOM *P*‐value	FDR *q*‐value	FWER *P*‐value
HALLMARK_ANGIOGENESIS	−0.391	−1.449	*P* < 0.001	0.676	0.241
HALLMARK_ADIPOGENESIS	−0.306	−1.432	*P* < 0.001	0.362	0.288
HALLMARK_ALLOGRAFT_REJECTION	−0.295	−1.402	*P* < 0.001	0.354	0.351
HALLMARK_APICAL_SURFACE	−0.445	−1.377	*P* < 0.001	0.376	0.452
HALLMARK_REACTIVE_OXYGEN_SPECIES_PATHWAY	−0.265	−1.370	*P* < 0.001	0.319	0.491
HALLMARK_ESTROGEN_RESPONSE_EARLY	−0.333	−1.366	*P* < 0.001	0.274	0.491
HALLMARK_IL6_JAK_STAT3_SIGNALING	−0.383	−1.361	*P* < 0.001	0.242	0.491
HALLMARK_TNFA_SIGNALING_VIA_NFKB	−0.312	−1.264	*P* < 0.001	0.300	0.739
HALLMARK_UV_RESPONSE_DN	−0.427	−1.256	*P* < 0.001	0.284	0.802
HALLMARK_INTERFERON_GAMMA_RESPONSE	−0.519	−1.245	*P* < 0.001	0.294	0.85
HALLMARK_INTERFERON_ALPHA_RESPONSE	−0.611	−1.244	*P* < 0.001	0.281	0.85
HALLMARK_XENOBIOTIC_METABOLISM	−0.237	−1.226	*P* < 0.001	0.313	0.903
HALLMARK_INFLAMMATORY_RESPONSE	−0.311	−1.223	*P* < 0.001	0.300	0.903
HALLMARK_HYPOXIA	−0.370	−1.221	*P* < 0.001	0.282	0.903
HALLMARK_COAGULATION	−0.422	−1.221	*P* < 0.001	0.267	0.903
HALLMARK_APICAL_JUNCTION	−0.434	−1.129	*P* < 0.001	0.420	1

### Validation of WNT target induction by AS1842856 treatment through qRT‐PCR

**Fig. 2:**
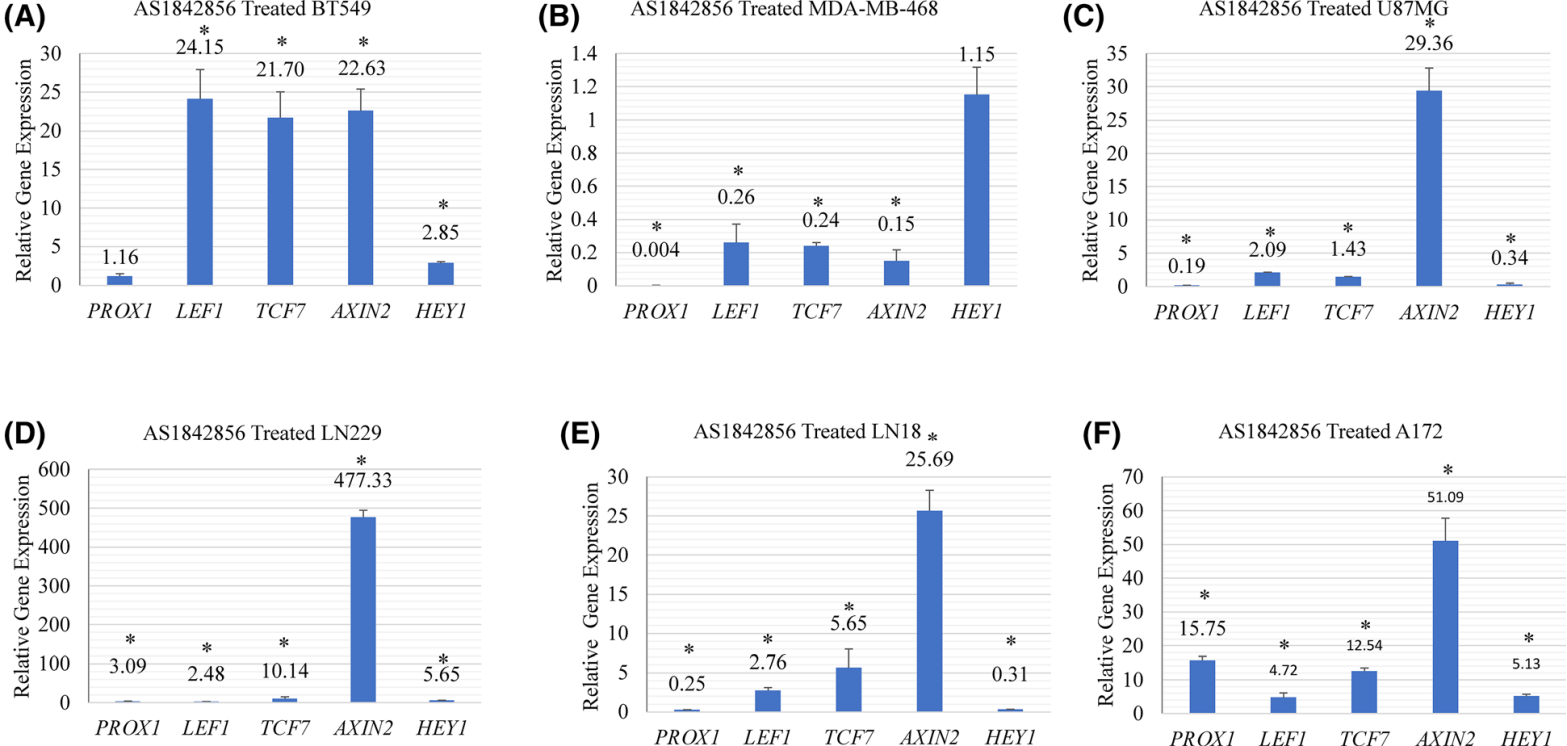
AS1842856 treatment induced canonical WNT target genes in a series of BBC and GBM cancer cell lines. Indicated cell lines were treated with 1 μm AS1842856 for 48 h and examined for changes in gene expression by qRT‐PCR using *TUBB* as the reference gene. AS1842856 treatment induced canonical WNT target genes in (A) BBC BT549 but not MDA‐MB‐468 BBC cells (B). (C–F) GBM cell lines had evidence of WNT target gene induction upon AS1842856 treatment; * denotes significantly different by Student's *t*‐test compared with the control (*P* < 0.05) with SD error bars. Each experiment had three biologically independent replicates.

**Fig. 3:**
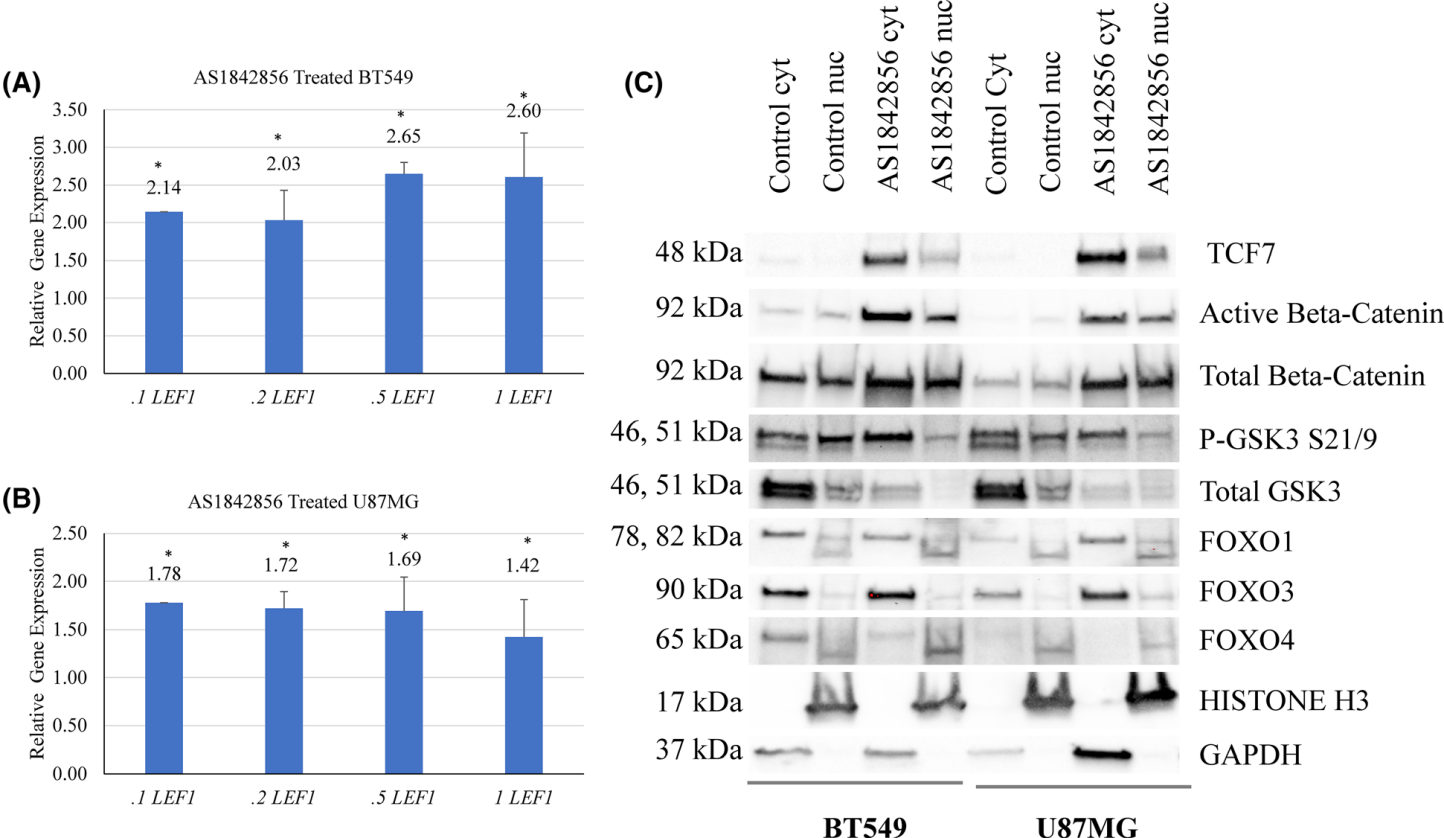
AS1842856 treatment increased beta‐catenin and decreased GSK3β protein expression. (A, B) Indicated BBC and GBM cell lines were treated with 0.1–1 μm AS1842856 for 48 h and examined for changes in gene expression by qRT‐PCR using *TUBB* as the reference gene. AS1842856 treatment increased *LEF1* gene expression. (C) BT549 and U87MG cells were treated with AS1842856 for 48 h, and then fractions were prepared; GAPDH and Histone H3 were cytoplasmic and nuclear controls respectively. Active and total beta‐catenin were increased, whereas GSK3β was decreased. * denotes significantly different by Student's *t*‐test compared with the control (*P* < 0.05) with SD error bars. Each experiment had three biologically independent replicates.

To validate differentially expressed WNT‐related genes obtained from RNA‐Seq data, we performed qRT‐PCR. Primers were designed using primer3 (v0.4.0) online tool (Table S1). WNT pathway genes *LEF1*, *TCF7*, *AXIN2*, and *HEY1* were increased upon AS1842856 treatment in BT549 cells (Fig. 2A). Validation of differential gene expression by qRT‐PCR built confidence in the RNA‐Seq analysis. We sought to examine whether FOXO1 inhibition would induce canonical WNT targets in another BBC cell line. None of the examined WNT genes were increased in BBC cell line MDA‐MB‐468 upon AS1842856 treatment (Fig. 2B). We investigated a set of GBM cell lines for changes in WNT pathway targets, given that this cancer also has FOXO‐regulated stem gene signatures [1, 23, 70]. Treatment with AS1842856 led to increased expression of *LEF1*, *TCF7*, and *AXIN2* in U87MG, LN229, LN18, A172, and DBTRG cells (Fig. 2C–F and Fig. S1A). *AXIN2* induction was particularly robust in these settings (26 to 477‐fold increased with AS1842856 treatment compared with control samples). *PROX1* and *HEY1* were induced by AS1842856 treatment in LN229 and A172 cells (Fig. 2D,F). *PROX1* was diminished in U87MG and LN18 cells upon AS1842856 treatment (Fig. 2C,E). Thus, *AXIN2*, *LEF1*, and *TCF7* were induced by AS1842856 treatment in six out of seven examined BBC and GBM cell lines. Experiments were done using 10‐fold less AS1842856 (samples ranging from 100 nm to 1 μm). Induction of *LEF1* was observed even with 100 nm of AS1842856 in BT549 and U87MG cells. Prior studies found that > 1 μm of AS1842856 was needed to efficiently inhibit FOXO3 or FOXO4, whereas 100 nm of the drug was sufficient to inhibit FOXO1 (Fig. 3A,B) [71, 81]. It is likely that FOXO3 and FOXO4 functionally overlap with FOXO1 in regulating WNT genes, as was observed in AML [63].

### AS1842856 treatment impacted WNT pathway genes in colon cancer cells

The WNT pathway is of central importance in colon cancer, with 80% having inactivating mutations in the *APC* gene that encodes a protein that promotes beta‐catenin destruction [35, 82, 83]. The WNT pathway sustains cancer stem cells and promotes the proliferation of cells as they migrate before differentiation [84, 85]. To ascertain the impact of *FOXO1* on WNT pathway gene expression in colon cancer, we treated HCT116 and SW480 cells with AS1842856. In the HCT116 cell line, *AXIN2*, *TCF7*, and *HEY1* were significantly induced upon AS1842856 treatment (Fig. S1B,C); *WNT7B* was also decreased in HCT116 cells (Fig. S1E). In the SW480 cell line, *HEY1* and *WNT5A* were slightly upregulated, whereas *WNT7B* was decreased upon AS1842856 treatment (Fig. S1C,F). Therefore, AS1842856 had varied impacts on colon cancer cell lines.

Secreted WNT ligands are implicated in breast cancer development. Studies indicated that *WNT5A* might have tumor suppressive roles, at least in some contexts, whereas *WNT5B* activates the WNT pathway in BBC [45, 46]. WNT7B expression was significantly associated with poor prognosis in breast cancer [86]. RNA‐Seq data revealed that several WNT‐secreted factors were differentially expressed upon AS1842856 treatment in BT549 cells. *WNT5A* was induced by treatment, whereas *WNT5B* and *WNT7B* were diminished, Table 3. These genes were analyzed in a set of BBC and GBM cell lines by qRT‐PCR. BT549 and LN18 had increased *WNT5A* and decreased *WNT5B* and *WNT7B* (Fig. S2A,E). MDA‐MB‐468 only had diminished *WNT5B* and *WNT7B* (Fig. S2B). U87MG and DBTRG cells had decreased *WNT7B* with AS1842856 treatment (Figs S1D and S2C). LN229 and A172 had increased *WNT5B* with AS1842856 treatment (Fig. S2D,F). Therefore, the impact of AS1842856 treatment on WNT ligands differed depending on the context in examined BBC and GBM cells.

**Table 3:** WNT ligand expression in BT549 RNA‐Seq data.

Gene	log_2_FoldChange (AS1842856/control)	*P*‐value	*P*‐adj
WNT5A	2.034	2.30E‐229	6.30E‐227
*WNT5B*	−0.592	8.00E‐97	6.51E‐95
*WNT7B*	−2.399	*P* < 0.001	*P* < 0.001

### AS1842856 treatment led to increased active and total beta‐catenin in BT549 and U87MG cells

We examined beta‐catenin subcellular localization and abundance to gain insight into why AS1842856 treatment led to increased target gene expression. Nuclear and cytoplasmic fractions were prepared for BT549 and U87MG cells treated with or without the drug. We found that the nuclear and cytoplasmic distributions of beta‐catenin were comparable, but the amount of total and active beta‐catenin was increased with AS1842856 treatment (Fig. 3C). GSK3β phosphorylates beta‐catenin leading to its degradation. GSK3β protein was reduced in AS1842856 treated samples, likely explaining the increase in beta‐catenin (Fig. 3C). We performed microscopy with AS1842856 treated cells to determine whether this drug impacted beta‐catenin membrane recruitment (using transfected beta‐catenin‐GFP). No change in beta‐catenin membrane recruitment was observed in AS1842856‐treated samples (Fig. S3).

### FOXO1 RNAi led to decreased canonical WNT gene expression

**Fig. 4:**
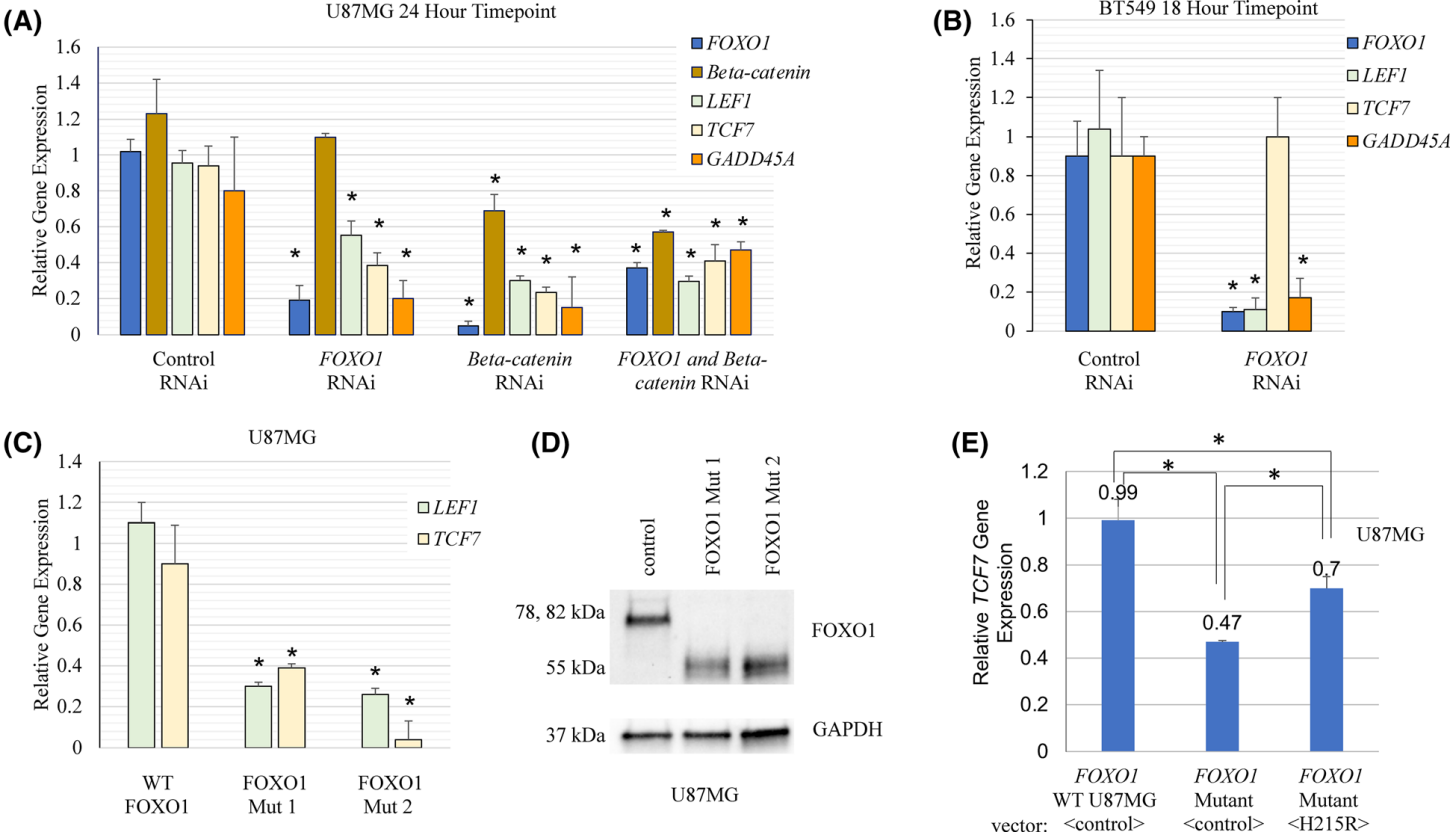
*FOXO1* RNAi‐targeting or disruption impacted WNT target gene expression. (A) *FOXO1* and/or beta‐catenin (*CTNNB1*) RNAi led to reduced *LEF1*, *TCF7*, and *GADD45A* gene expression 24 h post‐transfection in U87MG cells by qRT‐PCR using *TUBB* as the control. (B) *FOXO1* RNAi led to reduced *LEF*, and *GADD45A* gene expression 18 h post‐transfection in BT549 cells by qRT‐PCR using *TUBB* as the control. (C, D) *FOXO1* disruption by CRISPR Cas9 genome editing in U87MG cells led to reduced *LEF1* and *TCF7* gene expression measured by qRT‐PCR using *TUBB* as the control; disruption of FOXO1 produced a truncated protein as assessed by western blot analysis. The disruption mutant is predicted to express the C‐terminal portion of FOXO1 starting in the middle of the DNA‐binding domain. (E) Exogenous *FOXO1‐H215R* (DNA‐binding‐defective) significantly restored *TCF7* gene expression to *FOXO1* disruption mutants. * denotes significantly different by Student's *t*‐Test compared with the control (*P* < 0.05) with SD error bars. Each experiment had three biologically independent replicates.

To rigorously investigate the contribution of *FOXO1* to WNT gene regulation, we performed RNAi experiments. We found the reduction in *FOXO1* significantly decreased *LEF1* and *TCF7* gene expression (Fig. 4A,B). The *FOXO1* RNAi data contrast with results obtained with AS1842856 treatment, which induced WNT genes. To examine the role of FOXO1 in WNT gene regulation in another way, we built *FOXO1* disruption mutants using CRISPR Cas9 genome editing in U87MG cells (confirmed by western blot and sequencing). We found that *LEF1* and *TCF7* gene expression was decreased in *FOXO1* disruption mutants (Fig. 4C,D and Fig. S4). Exogenous expression of the DNA‐binding‐defective *FOXO1*‐H215R mutant significantly restored *TCF7* expression to *FOXO1* disruption mutants in the U87MG background (Fig. 4E). FOXO1 mutant H215R has a histidine that directly contacts DNA replaced with arginine. Numerous studies have validated that this mutant fails to bind tested target DNA sequences, including 3XIRS [72, 73]. Transfections of *FOXO1‐H215R* mutant were done via electroporation using a protocol for mouse neurons, given that these cells were difficult to transfect, similar to our previous work with *FOXO3* mutants in GBM cells [70]. Strikingly, it appears that the DNA‐binding activity of FOXO1 is dispensable for *TCF7* induction, possibly explaining why AS1842856 treatment induced *TCF7*, as this drug only impedes the ability of FOXO1 to directly bind to DNA. In support of this idea, a previously published *FOXO1‐H215R*‐induced target *TCF4* was also significantly increased by AS1842856 treatment in our RNA‐Seq data (*P* = 1.25E‐38) [73].

**Fig. 5:**
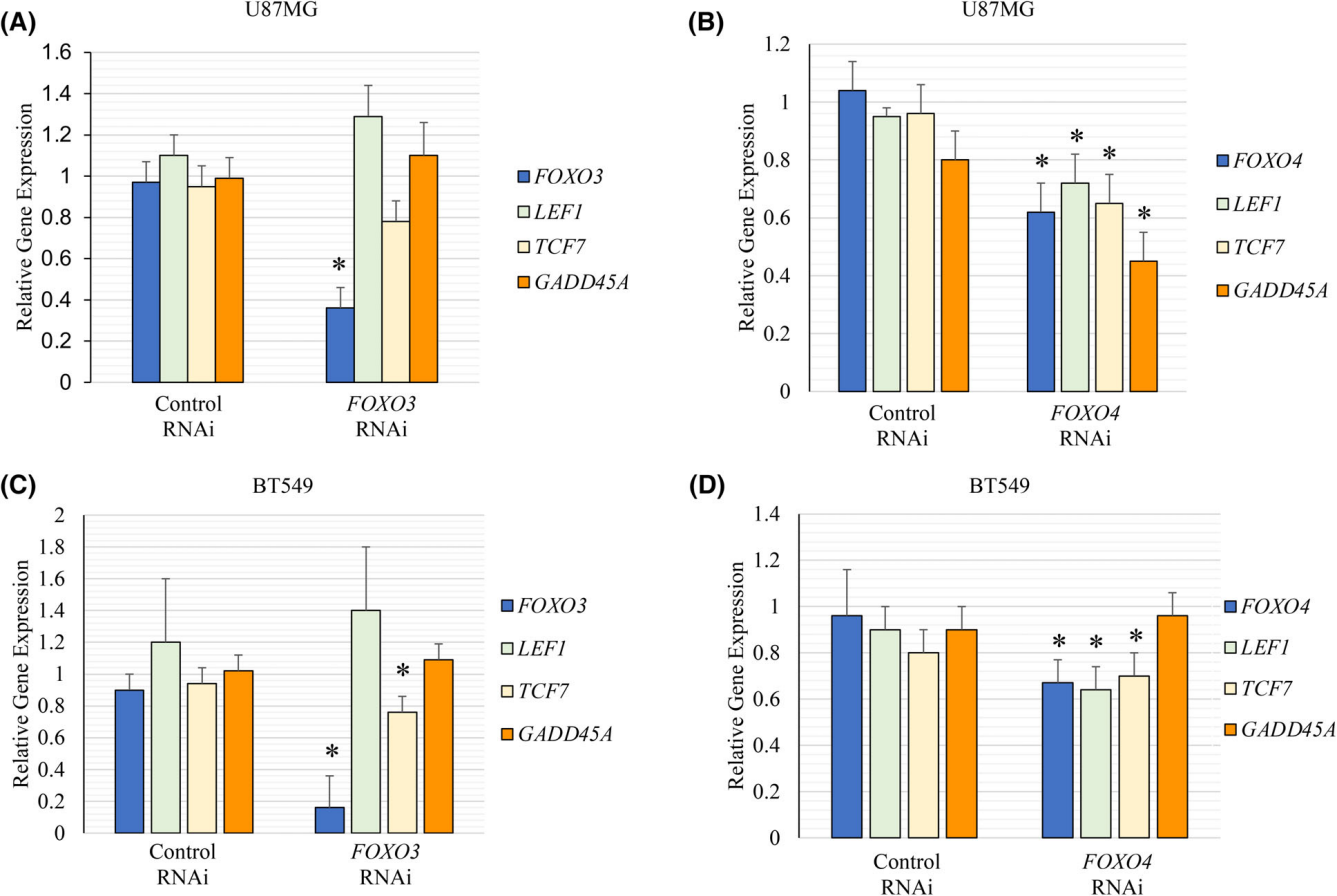
*FOXO3* and *FOXO4* RNAi‐targeting reduced WNT target gene expression. (A) *FOXO3* RNAi 24 h post‐transfection in U87MG cells was assessed by qRT‐PCR using *TUBB* as the control. *FOXO3* RNAi had no significant impact on *LEF1* or *TCF7*. (B) *FOXO4* RNAi was assessed 24 h post‐transfection U87MG cells and reduced *LEF1*, *TCF7*, and *AXIN2* by qRT‐PCR using *TUBB* as the control. (C, D) *FOXO3* or *FOXO4* RNAi was assessed 24 h post‐transfection by RT‐PCR using *TUBB* as the control in BT549 cells; *FOXO3* RNAi samples impacted *TCF7*, whereas *FOXO4* reduction samples had reduced *LEF1* and *TCF7*. * denotes significantly different by Student's *t*‐test compared with the control (*P* < 0.05) with SD error bars. Each experiment had three biologically independent replicates.

We performed RNAi experiments to examine contributions from *FOXO3* and *FOXO4* to WNT pathway gene expression (24‐h timepoint). We found that the reduction in *FOXO3* only reduced *LEF1* and *TCF7* in BT549 cells (not U87MG cells), whereas *FOXO4* RNAi led to reduced *LEF1* and *TCF7* in U87MG cells and reduced *LEF1* in BT549 cells (Fig. 5A–D). Therefore, FOXO1 promotes *LEF1* and *TCF7* gene expression in U87MG and BT549 cells with some functional overlap with FOXO3 and FOXO4.

### WNT targets were robustly overexpressed in GBM and colon cancer clinical samples

**Fig. 6:**
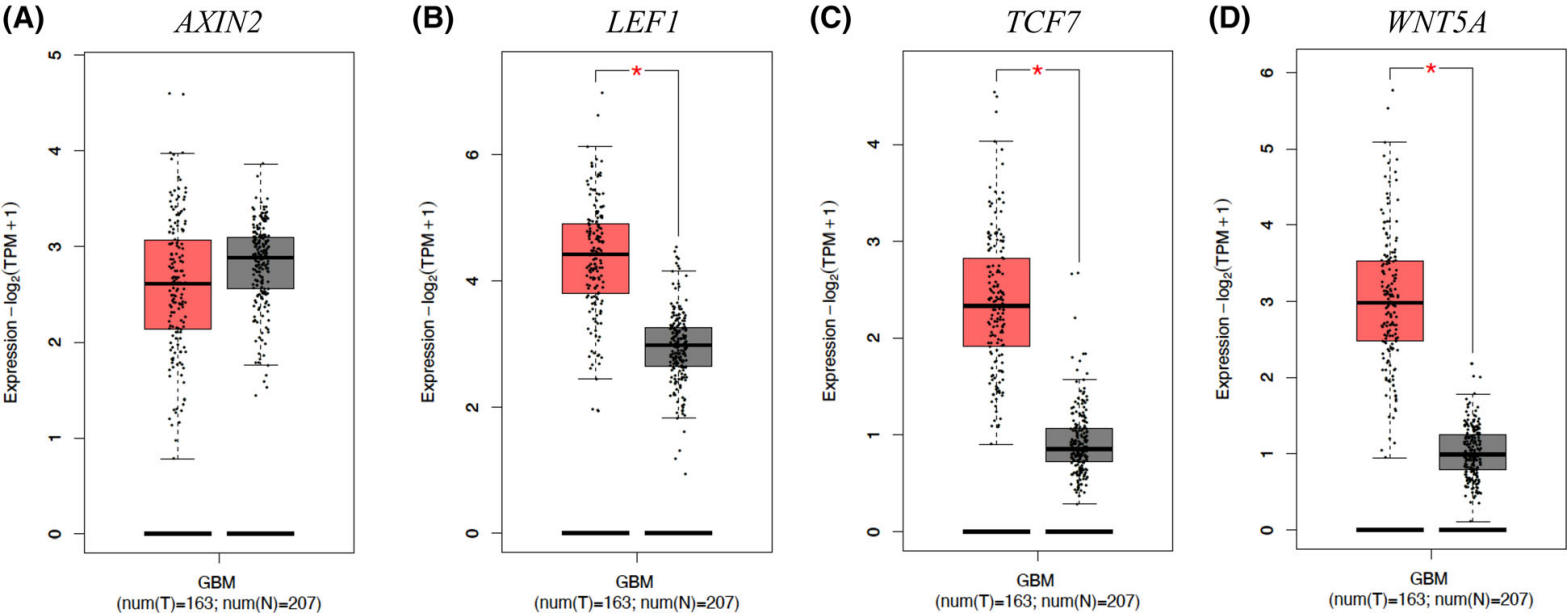
WNT genes are overexpressed in GBM clinical samples. (A–D) Differential expression boxplots of WNT pathway genes (*AXIN2*, *LEF1*, *TCF7*, and *WNT5A*) in GBM are shown. Data were from the Cancer Genome Atlas (TCGA) database using GEPIA2 and analyzed by one‐way ANOVA (*P* < 0.01).

**Fig. 7:**
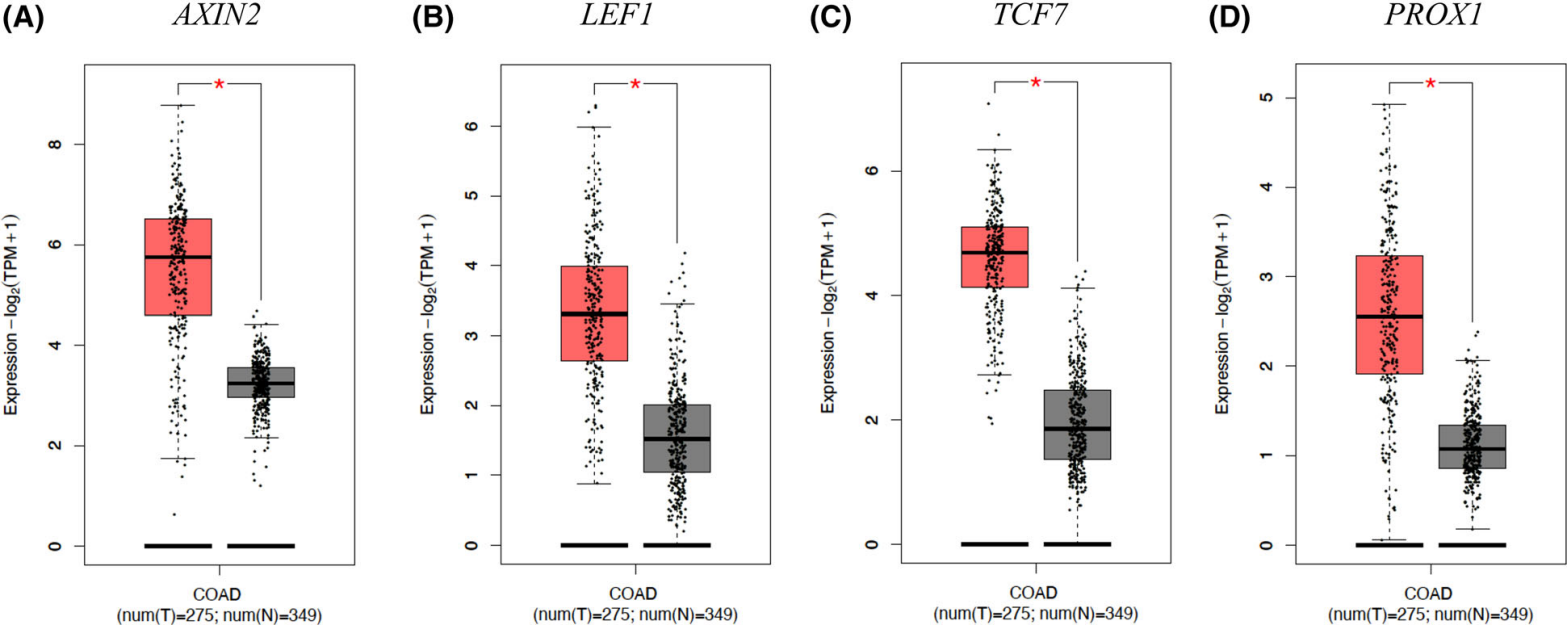
WNT genes are overexpressed in colon cancer clinical samples. (A–D) Differential expression boxplots of WNT pathway genes (*AXIN2*, *LEF1*, *TCF7*, and *PROX1*) in colon cancer (COAD) are shown. Data were from the Cancer Genome Atlas (TCGA) database using GEPIA2 and analyzed by one‐way ANOVA (*P* < 0.01).

The WNT pathway has many roles in cancer, including within stem and proliferating cells [46, 86]. To gain insight into WNT pathway expression in clinical samples, we queried the GEPIA2 database, which has gene expression and matched control clinical data [74]. *AXIN2*, *LEF1*, *TCF7*, and *WNT5A* were robustly increased in GBM compared with control samples (Fig. 6A–D). *AXIN2*, *LEF1*, *TCF7*, and *PROX1* were highly expressed in colon cancer compared with control samples (Fig. 7A–D). These results support the notion that canonical WNT gene expression is elevated and relevant in GBM and colon cancer clinical samples. Refining regulatory mechanisms that promote these WNT outputs, such as FOXO1‐mediated, will shed light on the biological underpinnings that drive GBM and colon cancer progression.

## Discussion

The WNT pathway facilitates stem cell renewal, cellular differentiation, and proliferation in cancer [32, 38, 45, 46, 85, 86]. Our data showed that FOXO1 promoted the expression of canonical WNT genes *LEF1* and *TCF7* in BT549 BBC and U87MG GBM cancer cells (Figs 3A,B and 4A,B). This work expands the repertoire of contexts in which *FOXO1* promotes canonical WNT gene expression to include BBC and GBM cells examined. Prior studies in *Drosophila*, T‐cell development, and murine AML models also found that FOXO factors promoted beta‐catenin‐driven gene expression [59, 63]. Our work is the first to highlight that FOXO factors promote canonical WNT gene expression in cancers derived from the breast and brain.

AS1842856 treatment induced canonical WNT genes, even in low dosages (Figs 1A,B and 2A,C–F). It appears that AS1842856 acts as a FOXO1 modulator instead of solely as an inhibitor. Differential impacts of AS1842856 treatment on FOXO1 target genes are possible, especially given its ability to specifically bind to only the unphosphorylated form of this protein to hinder DNA binding [71]. We posit that AS1842856 only inhibited the gene expression of a subset of FOXO1 targets that likely required DNA binding, such as *SOD2*, *INSR*, and *GADD45A* (*P*‐values 1.37E‐158, 3.54E‐08, and 1.27E‐21 respectively). The DNA‐binding function of FOXO1 was not required for it to induce *TCF7*, as evidenced by the ability of *FOXO1‐H215R* to restore its gene expression to *FOXO1* disruption mutants (Fig. 4E). These results might explain why AS1842856 (which blocks DNA binding) increased *TCF7*. Perhaps FOXO1 is released from the DNA by AS1842856 and is repurposed to targets that do not require direct DNA binding, such as *TCF7*. Future studies that employ chromatin immunoprecipitation experiments will shed light on this possibility.

There is precedence for DNA‐binding‐independent gene regulation by *FOXO1*. For example, *FOXO1‐H215R* induced the related WNT pathway component *TCF4* in renal carcinoma cells; this gene was also increased in our RNA‐Seq data by AS1842856 treatment (*P* = 1.25E‐38) [73]. A related Foxo1 DNA‐binding‐defective mutant with H212 mutated to R (murine model) induced *Hes1* in myoblasts where Foxo1 acted as a co‐activator that was brought to targets by the protein Csl (CBF1, suppressor of hairless, Lag‐1) [72]. Therefore, FOXO1‐H215R (and related mutant in mice) induces the expression of numerous transcription factors, including *TCF7*, *TCF4*, and *HES1*.

FOXO1 inhibition impacted *AXIN2*, *LEF1*, and *TCF7* in some, but not all, examined BBC and colon cancer cell lines. One possibility for this divergence could be that the WNT pathway is poised to activate gene expression in some contexts but not others. Perhaps, the distinct BBC subtype contributes to WNT pathway activity. BT549 cells are of the Basal B subtype, and MDA‐MB‐468 are Basal A. Moreover, beta‐catenin was observed in both the nucleus and cytoplasm of BT549 cells and in the cytoplasm of MDA‐MB‐468 cells, highlighting a possible mechanism for different WNT gene expression dynamics in these settings [87, 88]. Identifying the conditions that dictate WNT pathway regulation by FOXO1 in cancer will allow for a greater understanding of divergent responses to AS1842856 treatment in these settings.

FOXO factors were previously reported to negatively regulate WNT pathway targets such as *CCND1* in pancreatic and prostate cancers as well as osteosarcoma [66, 67, 89]. In osteosarcoma cells, FOXO1 activation led to decreased *CCND1* (encoding cyclin D1) expression [66]. Therefore, in some settings, FOXO factors hinder WNT and promote this pathway in others. One possible explanation for these striking differences is that FOXO output is highly context‐dependent [90, 91]. Perhaps, co‐factors and/or post‐translational modifications direct the ability of FOXO to promote WNT gene expression in AML and GBM cells, whereas another set of circumstances, such as under stress, direct FOXO to disrupt the WNT pathway in DLD1 and pancreatic cancer cells. The localization of FOXO factors may also impact their ability to regulate the WNT pathway. For example, FOXO3 did not affect *LEF1* and *TCF7* in U87MG cells and was primarily cytoplasmic in this setting (Figs 3C and 5A). Unraveling the contextual frameworks that dictate the impact of FOXO factors on the WNT pathway will allow for a greater understanding of the mechanisms that drive cancer progression.

## Conclusion

FOXO transcription factors are emerging as key regulators of the WNT pathway in settings ranging from osteoblasts to cancer. This work demonstrates for the first time that FOXO1 promotes the expression of WNT pathway target genes *LEF1* and *TCF7* in BT549 BBC and U87MG GBM cells. *FOXO1* RNAi targeting or disruption by CRISPR Cas9 genome editing led to reduced expression of these genes. Our work highlights caveats for utilizing AS1842856, as this drug modulates FOXO1 activity instead of merely inhibiting it. Based on our work, some FOXO1 targets were increased with AS1842856 treatment, such as *TCF7* and *TCF4*, and some were significantly reduced, such as *SOD2*, *INSR*, and *GADD45A*. Over 70 peer‐reviewed publications employed AS1842856 to identify FOXO1 targets and may need reassessment. There are increasing cases in which FOXO1 regulates transcription in a DNA‐binding‐independent manner, including published targets *TCF4* and *HES1*, as well as in our work regarding *TCF7* (Fig. 4E) [72, 73].

## Conflict of interest

The authors declare no conflict of interest.

## Author contributions

SP, AL, DF, BL, JN, BS, RP, RG, and MK performed experiments. SP, DF, AL, BS, MWP, RG, BG, and MK critically analyzed data. S.P. and M.K. wrote the manuscript.

## Supporting information


**Fig. S1.** FOXO1 inhibition induced WNT target genes in GBM DBTRG and HCT116 cancer cells.

**Fig. S2.** FOXO1 inhibition impacted WNT ligand gene expression in a context‐dependent manner.

**Fig. S3.** AS1842856 treatment did not impact beta‐catenin localization.

**Fig. S4.** Sequencing of *FOXO1* disruption mutant.

**Table S1.** PCR primers.

## Data Availability

All cell lines and additional data prepared from this work are available upon request. RNA‐Seq data were deposited to the NCBI Geo database (Accession: GSE179856).
