# Supplementary material for: FOXO1 promotes the expression of canonical WNT target genes in examined basal‐like breast and glioblastoma multiforme cancer cells
Source: FEBS Open Bio. 2023 Aug 28;13(11):2108–23. doi: 10.1002/2211-5463.13696 (PMC10626282; doi:10.1002/2211-5463.13696)
Supplement: Supplementary file 1 — Fig. S1. FOXO1 inhibition induced WNT target genes in GBM DBTRG and HCT116 cancer cells. [file FEB4-13-2108-s005.pdf]

Figure S1

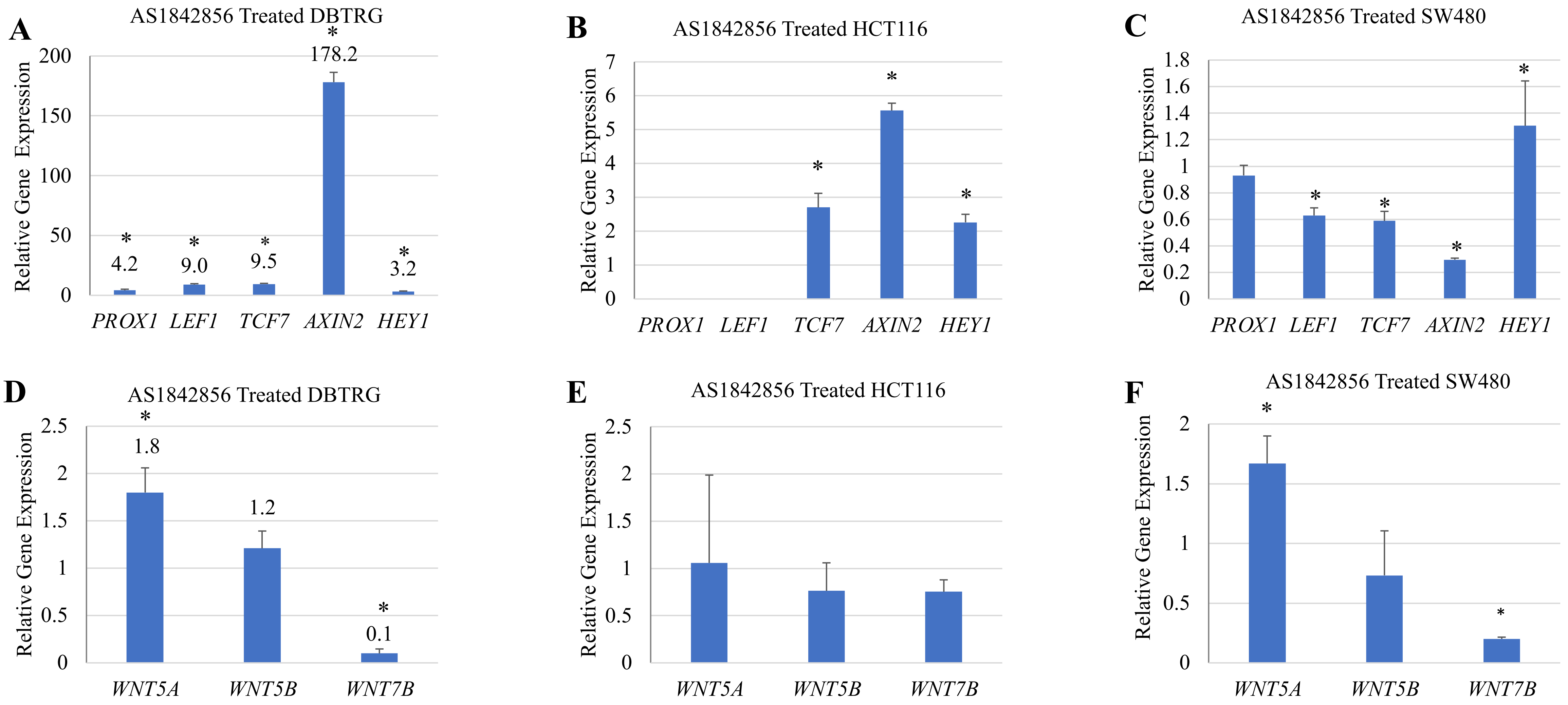

Figure S1: FOXO1 inhibition induced WNT target genes in GBM DBTRG and HCT116 colon cancer cells. Gene expression was assessed by qRT-PCR. **(A)** DBTRG cells were treated with 1µM AS1842856 for 48 hours (B) HCT116 colon cancer cells were treated with 1 µM AS1842856 for 48 hours **(C)** Colon cancer SW480 cells were treated with 1 µM AS1842856 for 48 hours **(D-F)**. Indicated cell lines were treated with AS1842856 for 48 hours and assessed by qRT-PCR. * denotes significantly different by Student's T-Test compared to the control (*P*<0.05) with SD error bars. Each experiment had three biologically-independent replicates.
