# Supplementary material for: FOXO1 promotes the expression of canonical WNT target genes in examined basal‐like breast and glioblastoma multiforme cancer cells
Source: FEBS Open Bio. 2023 Aug 28;13(11):2108–23. doi: 10.1002/2211-5463.13696 (PMC10626282; doi:10.1002/2211-5463.13696)
Supplement: Supplementary file 2 — Fig. S2. FOXO1 inhibition impacted WNT ligand gene expression in a context‐dependent manner. [file FEB4-13-2108-s003.pdf]

Figure S2

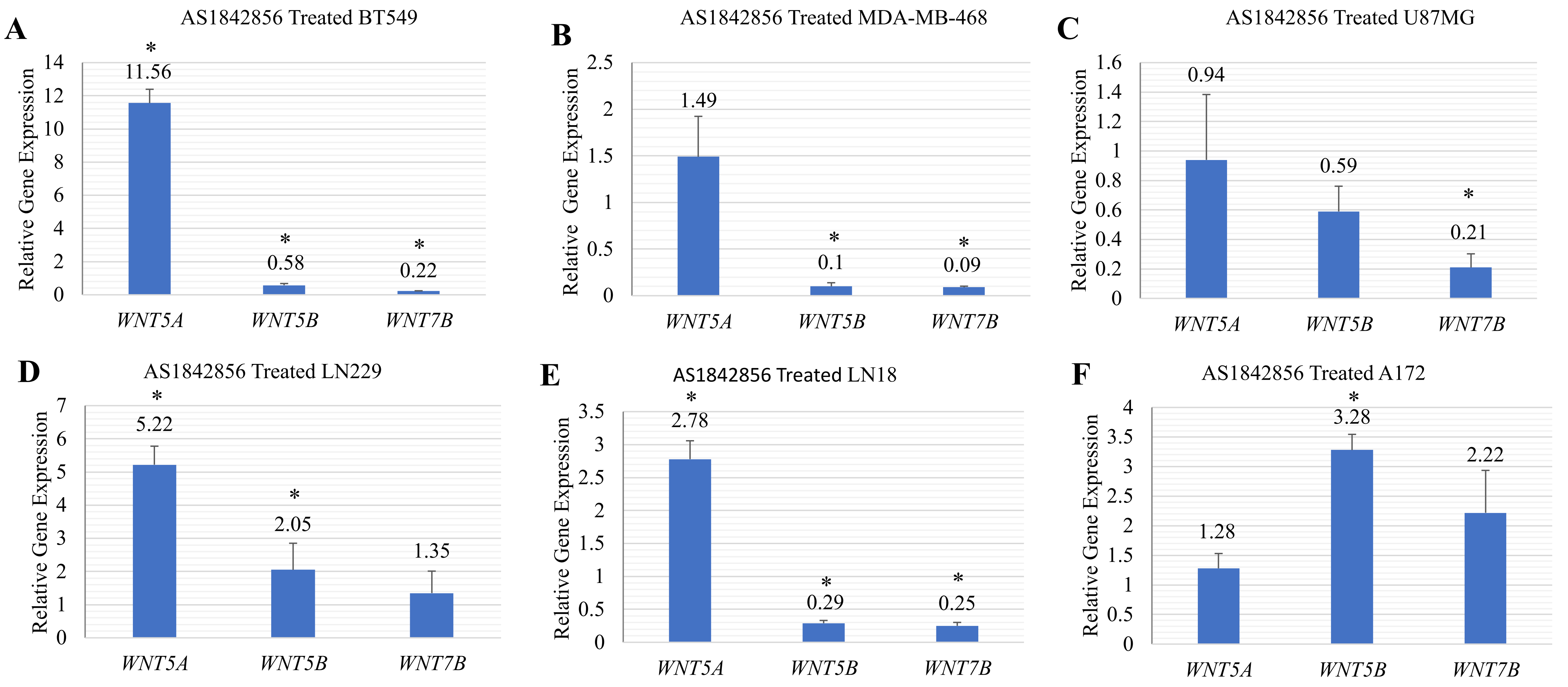

Figure S2: FOXO1 inhibition impacted WNT ligand gene expression in a context-dependent manner. Indicated cell lines were treated with 1 μM AS1842856 for 48 hours and examined for changes in gene expression by qRT-PCR using *TUBB* as the reference gene. **(A-F)** AS1842856 treatment impacted WNT ligand gene expression in a context-dependent manner. * denotes significantly different by Student's T-Test compared to the control ($P<0.05$) with SD error bars. Each experiment had three biologically-independent replicates.
