# Supplementary figures and images for: FOXO1 promotes the expression of canonical WNT target genes in examined basal‐like breast and glioblastoma multiforme cancer cells
Source: FEBS Open Bio. 2023 Aug 28;13(11):2108–23. doi: 10.1002/2211-5463.13696 (PMC10626282; doi:10.1002/2211-5463.13696)

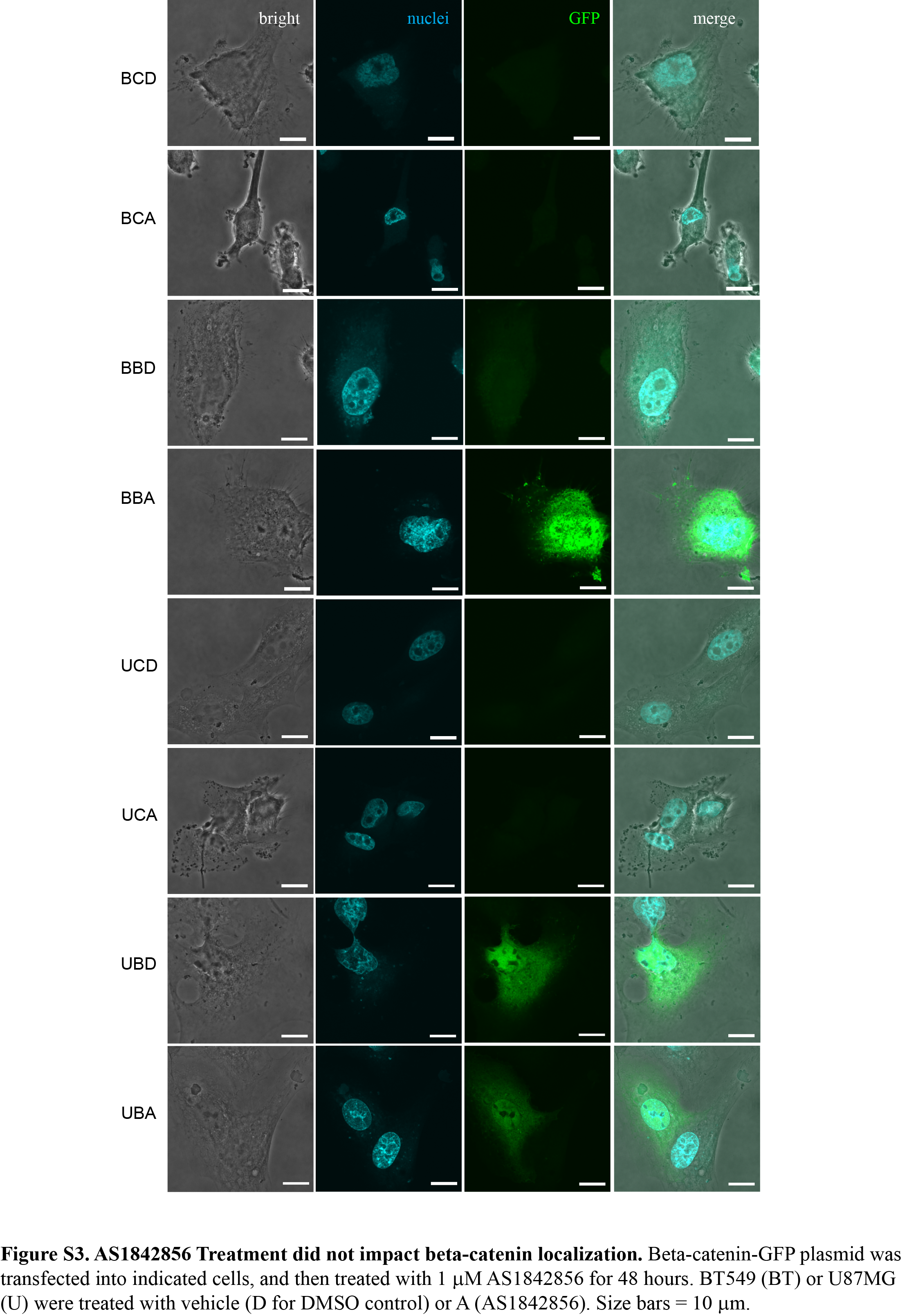

Supplement: Supplementary file 3 — Fig. S3. AS1842856 treatment did not impact beta‐catenin localization. [file FEB4-13-2108-s004.tif]
