# Supplementary material for: FOXO1 promotes the expression of canonical WNT target genes in examined basal‐like breast and glioblastoma multiforme cancer cells
Source: FEBS Open Bio. 2023 Aug 28;13(11):2108–23. doi: 10.1002/2211-5463.13696 (PMC10626282; doi:10.1002/2211-5463.13696)
Supplement: Supplementary file 4 — Fig. S4. Sequencing of FOXO1 disruption mutant. [file FEB4-13-2108-s001.pdf]

Figure S4

Sequence of *FOXO1* Disruption Mutant

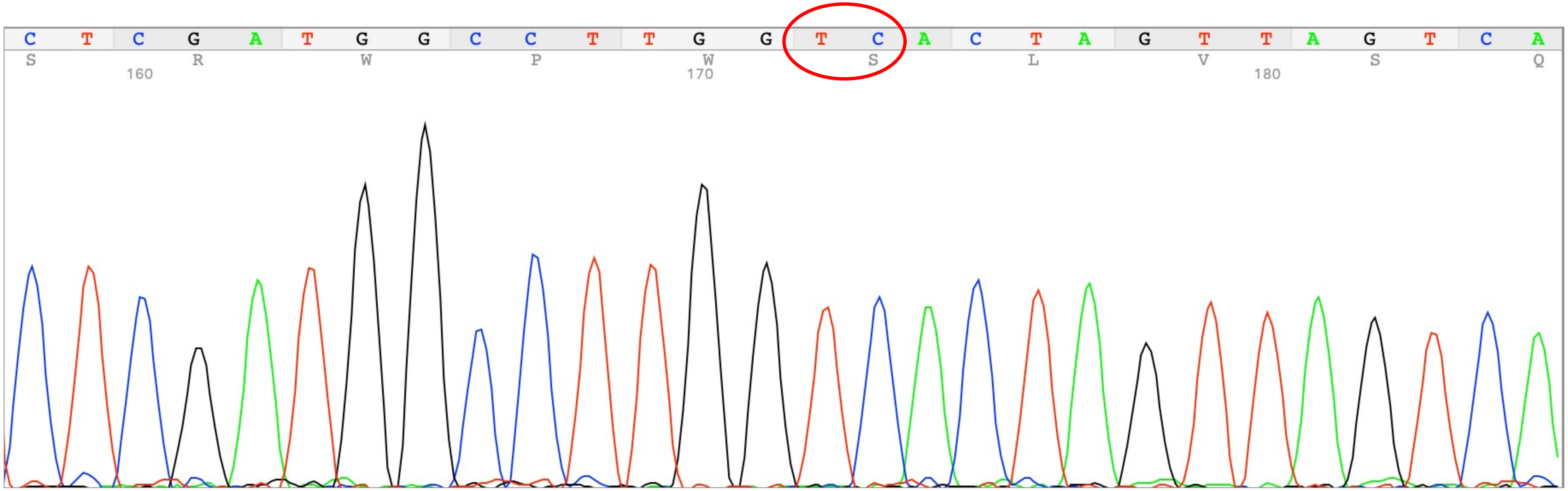

ccgagctctcgatggccttggtcactagttagtcaataacttcgtataat *FOXO1* mutant sequence

ccgagctctcgatggccttggtgatgaggtcggcgtaggacaggttgccc *FOXO1* genomic sequence

Figure S4. Sequencing of *FOXO1* disruption mutant.

The *FOXO1* gene was disrupted using CRISPR Cas9 genome editing technology in U87MG cells and sequenced. The red circle indicates the fusion site.
