# Supplementary material for: FOXO1 promotes the expression of canonical WNT target genes in examined basal‐like breast and glioblastoma multiforme cancer cells
Source: FEBS Open Bio. 2023 Aug 28;13(11):2108–23. doi: 10.1002/2211-5463.13696 (PMC10626282; doi:10.1002/2211-5463.13696)
Supplement: Supplementary file 5 — Table S1. PCR primers. [file FEB4-13-2108-s002.pdf]

Table S1. PCR Primers

Primer Name	Sequence (5' to 3')
AXIN2 F	TGTCCAGCAAAACTCTGAGG
AXIN2 R	TGGCTGGTGCAAAGACATAG
HEY1 F	CGCCCTTGCTATGGACTATC
HEY1 R	TTGTTGAGATGCGAAACCAG
LEF1 F	GGAAGGCGATTTAGCTGACA
LEF1 R	GTGTTCTCTGGCCTTGTCGT
PROX1 F	TTGGCAGGCCTACTATGAGC
PROX1 R	TTTTCATTGCCCCTTAATGC
TCF7 F	CCCCCAACTCTCTCTCTACG
TCF7 R	GGAGTAGAAGCCAGAGAGGTCA
TUBB F	CTGGACCGCATCTCTGTGTA
TUBB R	ATCTGGCCAAAAGGACCTG
WNT5A F	GCCCAGGTTGTAATTGAAGC
WNT5A R	TGAGAAAGTCCTGCCAGTTG
WNT5B F	GTTCACGGCTGCTCTGCT
WNT5B R	GGAAGCTGACTGCACACG
WNT7B F	ACGCTGGAACTGCTCTGC
WNT7B R	TTGCTCAGGTTCCCTTGG
GADD45A F	GGAAAGTCGCTACATGGATCA
GADD45A R	TTCATTCAGATGCCATCACC
5R (for *FOXO1* disruption genotyping)	CTTCTCTAGGCACCGGTTCA
FOXO1 R for genotyping	ACCTTCAGGCCGAGCAAAC
